# Usefulness of Safinamide in Lewy Body Dementia

**DOI:** 10.1002/alz.089827

**Published:** 2025-01-09

**Authors:** Antonio Oliveros‐Cid, Lisbed Rondon Moreno, Esther Sierrra Martinez, Pablo Navarro Beltran, Nicolas Fayed Miguel, Maria Angeles Cid Lopez, Fernando Jarauta Salvador, Antonio Oliveros‐Juste

**Affiliations:** ^1^ Hospital Reina Sofia, Tudela, Navarra Spain; ^2^ Fundacion Neuropolis, Zaragoza, Zaragoza Spain; ^3^ Hospital Viamed Montecanal, Zaragoza, 50012 Spain; ^4^ Hospital San Juan de Dios, Zaragoza, ZARAGOZA Spain; ^5^ Fundacion Neuropolis, Zaragoza Spain; ^6^ Hospital Universitario Miguel Servet, Zaragoza, Zaragoza Spain; ^7^ Hospital Quiron Zaragoza, Zaragoza, Zaragoza Spain; ^8^ Hospital Reina Sofia, Tudela de Navarra, Navarra Spain

## Abstract

**Background:**

The therapeutic management of dementia with Lewy bodies (LBD) is a challenge given the high sensitivity to drugs in this disease. This is particularly sensitive with regard to the management of parkinsonism. In particular, treatment of motor symptoms with levodopa or dopaminergic agonists poses a risk of worsening cognitive and behavioral symptoms. Safinamide is a drug with several mechanisms of action (dopaminergic and non‐dopaminergic), authorized to treat idiopathic Parkinson’s disease, complementary to levodopa alone or in combination with other antiparkinsonian drugs, in patients with fluctuations. The scarce literature on the use of safinamide in MCI shows benefits with few side effects.

**Method:**

We present 4 cases of patients with Dementia with Lewy Bodies (DLB) evaluated in our clinics, where safinamide improved motor symptoms without worsening cognitive or behavioral symptoms. MDS‐UPDRS (I, II, III, IV), RBDSQ, CGI‐S, CGI‐C, SEADL, PGI‐S, PGI‐C, MDS‐NMS, MoCA, CDT, RBANS were performed before and after at least 3 months of Safinamide therapy.

**Result:**

The study included 3 males and 1 female aged between 63 and 78 years, diagnosed with DLB 1 to 4 years before starting Safinamide treatment. The most evident parkinsonian symptoms were axial rigidity, antecollis, camptocormia, simple and complex motor fluctuations, and freezing of gait. All of them exhibited fluctuations in both motor and cognitive evolution, with characteristic cognitive decline and visual hallucinations. All had previously received treatment with L‐dopa and were on acetylcholinesterase inhibitor therapy. UPDRS‐III scores ranged from 24 to 43 points. Initial safinamide dose: 50 mg/24 h. Final safinamide dose: 100 mg/24 h. Mean improvement in UPDRS: 17%.

**Conclusion:**

In these 4 isolated cases, we observe that safinamide treatment improved axial and appendicular rigidity without worsening cognitive or behavioral symptoms. It could be considered as a safe and effective option in DLB, even in patients with insufficient improvement with L‐dopa, and without clinically significant interactions with acetylcholinesterase inhibitors. Further extensive studies would be necessary to confirm this preliminary observation.

